# Amoebic toxic megacolon with poly-helminthic coinfection: Case presentation and review of intestinal polyparasitic infections

**DOI:** 10.1016/j.ijscr.2020.04.032

**Published:** 2020-05-11

**Authors:** S. Yusof, Y. Zhao, J. Quah, C.E. Ernest Eu, L.M. Wang

**Affiliations:** a Department of General Surgery, Changi General Hospital, 2 Simei Street 3, S529889, Singapore; b Department of Pathology, Changi General Hospital, 2 Simei Street 3, S529889, Singapore

**Keywords:** Toxic megacolon, Intestinal parasitic infections, Case report

## Abstract

**Introduction:**

Intestinal parasitic infections are a health burden to developing countries, but can also become more prevalent worldwide, even in developed countries, with the advent of globalization. While most of these infections are benign, some may be associated with acute infections with high morbidity and mortality rates.

**Case presentation:**

A 36 years old patient presented with toxic megacolon with poly-helminthic infections, likely a result of raw food ingestion. She subsequently required multiple surgeries with a total colectectomy and small bowel resection, and ended up with an end-ileostomy.

**Discussion:**

Intestinal polyparasitism has been reported in undeveloped countries, but it appears that such a trend can be found in developed countries as well. Clinicians, especially those practicing in countries with large expatriate or immigrant worker populations, should be aware of this trend and adjust treatment protocols accordingly.

**Conclusion:**

Even in countries whereby intestinal parasitic infections are not common, clinicians should have a heightened awareness of the possibility for such infections to be present, especially in returning travellers, expatriate or immigrant populations.

## Highlights

• Intestinal parasitic infections are likely going to be increasingly common even in developed countries.
• While most of the infections are mild, severe cases can result in high morbidity and mortality.
• Primary care physicians seeing patients with risk factors for intestinal parasitic infections should be vigilant.
• If the clinical suspicion is high, physicians should consider further investigations or empirical anti-parasitic treatment

## 1 Introduction

Intestinal parasitic infections remain a significant public health burden for many rural areas and developing countries in the tropical and sub-tropical regions [[1], [2], [3], [4], [5], [6], [7], [8], [9], [10], [11]]. While most infections are insidious in nature and can be silent, they can also present acutely with severe disease associated with high morbidity and mortality [12]. Given the advent of globalisation and an ever-increasing migrant workforce, the prevalence of intestinal parasitic infections can become more common, even in developed countries [13]. As gastrointestinal complaints remain one of the more commonly noted presenting complaints to the primary care physician [14,15], the clinician, especially one practising in developed countries, should now have a heightened vigilance to the possibility of intestinal parasitic infections being a differential to the more commonly encountered viral gastroenteritis.

We present a case of amoebic toxic megacolon with helminthic coinfection in a 36 year-old patient. This work has been reported in line with the SCARE criteria [16].

## 2 Case introduction

A 36 years old Filipino woman domestic helper who had been working in Singapore for the past 2 years presented to the Accident and Emergency Medicine Department with a 10-day history of non-bloody diarrhoea, abdominal distension, altered mental state and fever. Prior to her admission she had a history of chronic abdominal pain with altered bowel habits which was treated symptomatically by her general practitioner. The patient was from a rural village in the Philippines and frequently ate pork liver prepared in vinegar, a Filipino dish “Kilawin” which includes raw fish, shellfish, and half-cooked meat mixed with vinegar and Ilocano delicacies “Papaitan, Isaw and Igado” which contain pig intestines.

**Fig. 1:**
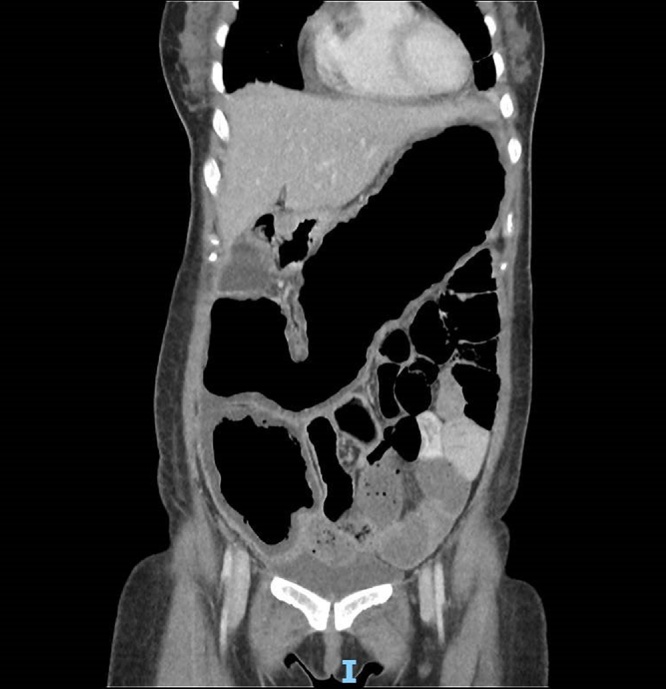
CT scan of dilated colon.

**Figs. 2–4:**
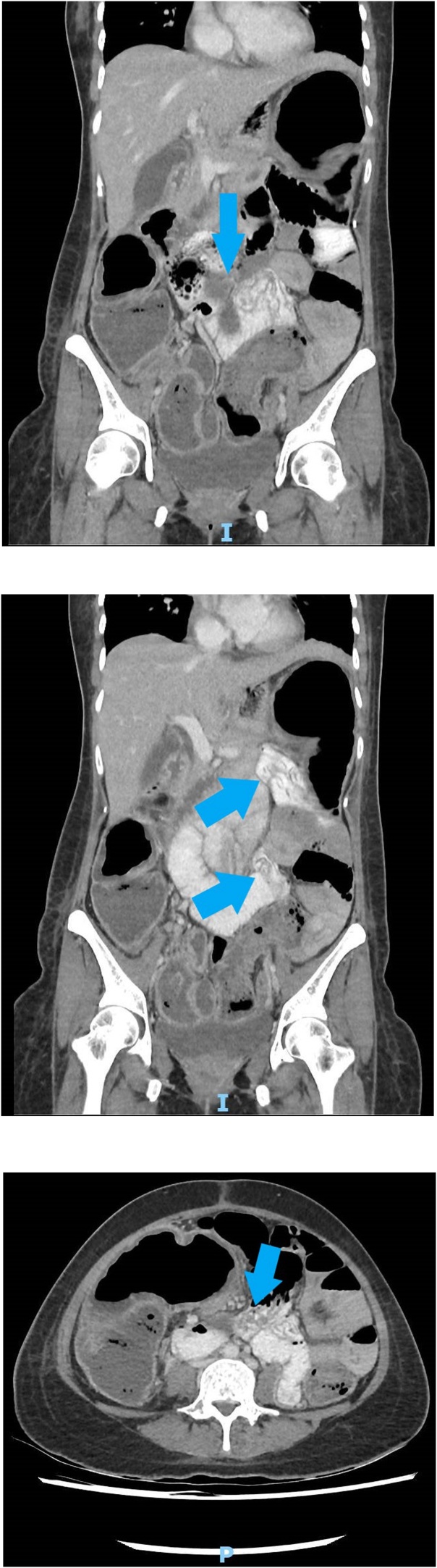
CT scan of possible small bowel worm infestation.

She was initially admitted with the diagnosis of gastroenteritis. However, her condition deteriorated with worsening abdominal pain and distension. An urgent surgical consult was sought following a computed tomography scan of the abdomen and pelvis which demonstrated grossly dilated colon suspicious for toxic megacolon with possible worm infestation of the small bowel (Fig. 1, Figs. 2–4). Her lab results on admission showed an elevated white cell count (14.6 × 10^3^/uL) with elevated C-reactive protein levels at 288.1 mg/L. Clinically, she was septic with signs of generalised peritonitis.

**Fig. 5:**
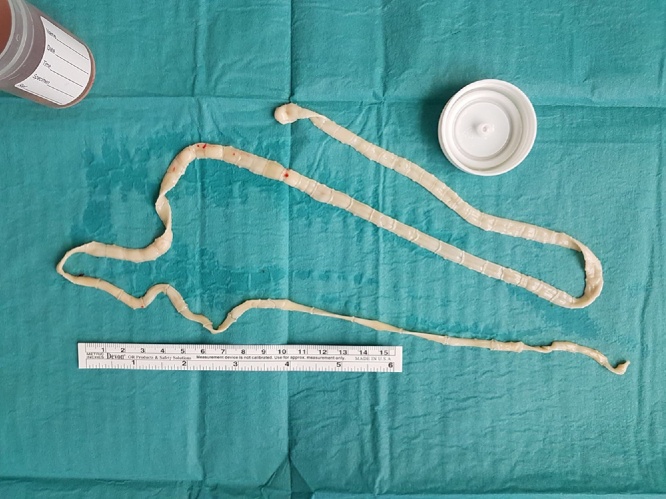
Adult tapeworm retrieved intraoperatively.

An emergent laparotomy performed revealed grossly dilated colon with numerous areas of ulceration and perforation resulting in faeculant peritonitis. A total colectomy was performed and during the transection of small bowel, segments of an adult tapeworm was retrieved (Fig. 5). As she was haemodynamically unstable on dual vasopressors with areas of small bowel ischaemia, decision was made for temporary abdominal closure. She subsequently underwent a relook laparotomy where a 2nd adult tapeworm was retrieved. As her rectal mucosa was ulcerated, an end ileostomy was formed.

She was commenced on IV metronidazole and oral albendazole and praziquantel. A Computed tomography scan of the brain was also performed in view of her history of altered mental state, but this did not reveal intracerebral lesions to suggest parasitosis or neurocysticerosis. She made a full recovery and was able to return to the Philippines.

Histopathology analysis of the total colectomy specimen showed there was fulminant amoebic (*Entamoeba histolytica*) colitis resulting in ulceration and perforation of the colon. In the specimens, co-helminthic infection with *Trichuris trichuira* nematodes and *Taenium solium cestodes* were also found.

## 3 Discussion

The World Health Organisation estimates that up to 50 million individuals are afflicted with *E. histolytica* and approximately 100 000 succumb to invasive amoebiasis annually, making the 3^rd^ leading cause of parasitic death, after malaria and schistosomiasis [11,17]. The outcome of infection with *E. histolytica* lies on a continuous spectrum, ranging from asymptomatic colonisation, to diarrhoea, colitis, presence of liver abscesses [18,19].

Although people can be asymptomatic from *E. Histolytica* infection, about 10–25% of patients develop gastrointestinal symptoms as the trophozoites invade the intestinal mucosa. Symptoms commonly expected by individuals include abdominal pain, diarrhoea (bloody, watery or mucoid). Diarrhoea can be as frequent as 10 or more bowel movements a day and fever occurs in 30% of patients [20]. Unfortunately there is a significant overlap of symptoms with other forms of bacterial dysenteries (*Salmonella*, *Campylobacter and E. Coli*) and shigellosis [21] which are endemic in the tropical and sub-tropical countries [22]. It is also worthwhile considering the diagnosis of non-infectious causes such as ischaemic colitis and inflammatory bowel disease.

Common diagnostic tests include faecal analysis which may reveal the presence of blood, lack of faecal leucocyte and the presence of Charcot-Leyden crystals. Colonoscopic findings vary from mucosal thickening to flask shape ulceration predominantly in the right side of the colon and rarely in the rectosigmoid area [23]. The best diagnostic test is detection of *E. Histolytica* antigen or DNA in faeces [24].

Intestinal polyparasitism is a phenomenon that has been reported in undeveloped regions including Brazil, Kenya, Congo, Colombia and Ivory Coast. Positive associations between *Schistosoma mansoni*, *Plasmodium falciparum* and hookworms in the Ivory Coast [25], as well as, *Trichuris* and *Ascaris lumbricoides* in Brazil [26] have been described in these population studies with stool analysis. In rural Malaysia, up to 71.4% of school children were found to have polyparasitism. *Trichuris*, *Ascaris*, hookworm, *Giardia duodenalis*, *Entamoeba* and *Cryptosporidium* species were amongst those identified [8,9]. Similar findings were reported amongst Kenyan school children with the inclusion of *Schistosoma mansoni* infections [27]. From the Ivory Coast study, it was revealed that three quarters of the studied population harboured at least three parasites concurrently, including high prevalence rate of many intestinal commensals such as *Entamoeba coli*, *Blastocystis hominis*, *Entamoeba hartmanni*, *Iodamoeba butschlii*, *Chilomastix mesnili* and *Endolimax nana* [28]. In Beira, Mozambique, it was found that 96% of the population harboured at least one helminth and that almost half (49%) harboured three helminths or more. The common species included *Strongyloides stercoralis* (48%), *Ancylostoma* spp. (25%) and *Necator americanus* (15%). A study by Boggild et al. in 2006 [13] on returning Canadian travellers and immigrants found that immigrants were more likely (OR 3.1) to be diagnosed with several intestinal protozoa (e.g. *E. Histolytica*, *Giardiasis*) than other travellers. The same study also found that travellers to certain Asian countries were at higher risks of acquiring intestinal parasitic infections, such as Pakistan, India, China and Bangladesh.

## 4 Conclusion

In conclusion clinicians, especially primary care physicians, practicing in countries with large expatriate and immigrant worker population such as Singapore should be suspicious of parasitic infections in patients with prolonged abnormal gastrointestinal symptoms, particularly in patient groups with the appropriate risk factors. A stool microscopy analysis may be insufficient to make a full diagnosis in the face of numerous parasite populations and ancillary parasite antigen testing can be considered. It may also be reasonable to start empirical therapy for immigrant worker populations as per the guidelines put forth by the World Health Organisation in 2017.

## Declaration of Competing Interest

There are no conflicts of interest.

## Sources of funding

There are no sources of funding for this research.

## Ethical approval

Because this is a case report, the present study was not appreciated by a research ethics committee. However, written informed consent was obtained from the patient for publication of this case report.

## Consent

Written informed consent was obtained from the patient for publication of this case report and accompanying images. A copy of the written consent is available for review by the Editor-in-Chief of this journal on request.

## Author contribution

S. Yusof: conceptualization, methodology, writing – review & editing, resources, supervision.

Zhao Y: investigation, writing – original draft, writing – review & editing.

J Quah: resources, writing – original draft.

Ernest Eu CE: writing – review & editing.

Wang LM: conceptualization, supervision.

## Registration of research studies

The present study is not a research involving humans, but a clinical case report, whose patient authorized the publication by means of a free and informed consent term.

## Guarantor

S Yusof, Zhao Y, Wang LM.

## Provenance and peer review

Not commissioned, externally peer-reviewed.
